# Could Heat Therapy Be an Effective Treatment for Alzheimer’s and Parkinson’s Diseases? A Narrative Review

**DOI:** 10.3389/fphys.2019.01556

**Published:** 2020-01-10

**Authors:** Andrew P. Hunt, Geoffrey M. Minett, Oliver R. Gibson, Graham K. Kerr, Ian B. Stewart

**Affiliations:** ^1^School of Exercise and Nutrition Sciences, Faculty of Health, Queensland University of Technology, Brisbane, QLD, Australia; ^2^Institute of Health and Biomedical Innovation, Queensland University of Technology, Brisbane, QLD, Australia; ^3^Centre for Human Performance, Exercise and Rehabilitation, College of Health and Life Sciences, Brunel University London, Uxbridge, United Kingdom; ^4^Division of Sport, Health and Exercise Sciences, Department of Life Sciences, College of Health and Life Sciences, Brunel University London, Uxbridge, United Kingdom

**Keywords:** neurodegenerative disease, heat shock protein, passive heating, thermal therapy, body warming, alpha-synuclein

## Abstract

Neurodegenerative diseases involve the progressive deterioration of structures within the central nervous system responsible for motor control, cognition, and autonomic function. Alzheimer’s disease and Parkinson’s disease are among the most common neurodegenerative disease and have an increasing prevalence over the age of 50. Central in the pathophysiology of these neurodegenerative diseases is the loss of protein homeostasis, resulting in misfolding and aggregation of damaged proteins. An element of the protein homeostasis network that prevents the dysregulation associated with neurodegeneration is the role of molecular chaperones. Heat shock proteins (HSPs) are chaperones that regulate the aggregation and disaggregation of proteins in intracellular and extracellular spaces, and evidence supports their protective effect against protein aggregation common to neurodegenerative diseases. Consequently, upregulation of HSPs, such as HSP70, may be a target for therapeutic intervention for protection against neurodegeneration. A novel therapeutic intervention to increase the expression of HSP may be found in heat therapy and/or heat acclimation. In healthy populations, these interventions have been shown to increase HSP expression. Elevated HSP may have central therapeutic effects, preventing or reducing the toxicity of protein aggregation, and/or peripherally by enhancing neuromuscular function. Broader physiological responses to heat therapy have also been identified and include improvements in muscle function, cerebral blood flow, and markers of metabolic health. These outcomes may also have a significant benefit for people with neurodegenerative disease. While there is limited research into body warming in patient populations, regular passive heating (sauna bathing) has been associated with a reduced risk of developing neurodegenerative disease. Therefore, the emerging evidence is compelling and warrants further investigation of the potential benefits of heat acclimation and passive heat therapy for sufferers of neurodegenerative diseases.

## Introduction

Humans are homeothermic and as such regulate their core body temperature within a narrow range. Perturbations to this homeostasis, induced by external environmental thermal stress or internally generated metabolic heat, produces both autonomic and behavioral responses designed to elicit a return of core body temperature toward thermal balance (Schlader and Vargas, 2019). While in an acute sense this stress response is a defense mechanism, regularly challenging the thermal equilibrium via active or passive thermal stress results in positive physiological and perceptual adaptations (Tyler et al., 2016). Recent research has shown positive therapeutic effects of passive heating for people with peripheral arterial disease (Neff et al., 2016; Akerman et al., 2019), chronic heart failure (Kihara et al., 2002; Ohori et al., 2012), diabetes (Hooper, 1999), and depression (Janssen et al., 2016). Passive heating also improves a range of health markers, including cardiovascular health indices, such as vascular function, blood pressure, and arterial stiffness (Brunt et al., 2016a, b), as well as metabolic health and glycemic control (Janssen et al., 2016; Kimball et al., 2018; Ely et al., 2019; Maley et al., 2019). Several mechanistic pathways may underpin these adaptations, including improved cellular respiration (Hafen et al., 2018), circulating factors (Brunt et al., 2019), and vascular shear stress (Tinken et al., 2009; Thomas et al., 2016). The upregulation of heat shock proteins (HSPs) as a result of acute and/or chronic (repeated) exposure to passive heating is also an adaptive outcome, which may provide a specific mechanistic pathway for improving health and function within the body (Faulkner et al., 2017; Brunt et al., 2018).

Recent reviews have identified the upregulation of HSPs as therapeutic targets for the treatment of neurodegenerative diseases including Parkinson’s disease and Alzheimer’s disease (Carman et al., 2013; Kalmar et al., 2014; Schapira et al., 2014; Ciechanover and Kwon, 2017; Webster et al., 2017; Klaips et al., 2018). Neurodegenerative diseases are characterized by the progressive deterioration of structures within the central nervous system responsible for motor control, cognition, and autonomic function. Alzheimer’s and Parkinson’s diseases are among the most common neurodegenerative diseases and have an increasing prevalence over the age of 50 (Pringsheim et al., 2014). Loss of protein homeostasis, due to protein mis-folding and aggregation of damaged proteins, is a hallmark of both Alzheimer’s and Parkinson’s diseases (Labbadia and Morimoto, 2015). HSPs function as chaperones to ensure appropriate cell function with distinct roles in the unfolded protein response, recognizing misfolded or mis-localized proteins that may be subsequently degraded by the proteasome, and are a key component of chaperone-mediated autophagy (Adachi et al., 2009; Stetler et al., 2010; Leak, 2014; Zarouchlioti et al., 2018). For their role in regulating protein homeostasis, HSP expression has been proposed as a therapeutic target for the treatment of these neurodegenerative diseases (Carman et al., 2013; Kalmar et al., 2014; Schapira et al., 2014; Ciechanover and Kwon, 2017; Webster et al., 2017; Klaips et al., 2018).

As physical and cognitive ability decline in Alzheimer’s and Parkinson’s diseases, passive heat therapy may yield an achievable alternative to the presently recommended exercise interventions in this population. Intriguingly, the incidence of Alzheimer’s disease has recently been shown to be reduced in people who undertook moderate to frequent sauna bathing (Laukkanen et al., 2017). While the current evidence for heat therapy in neurodegenerative disease is associative and the mechanisms by which improved health outcomes are achieved have yet to be elucidated, the potential of passive heating in this population remains an alluring therapeutic option.

This review will examine pathophysiological determinants of common neurodegenerative disease, examine the evidence of an elevated HSP expression as a potential therapeutic intervention in common neurodegenerative diseases, and describe the role heat acclimation and passive heat therapy have in inducing HSP expression. In addition, central and peripheral adaptations to body warming in healthy adults, including improved muscular function, cerebral blood flow, and metabolic health, will be considered with their potential influence on neurodegenerative disease outcomes. Finally, considerations for undertaking heat acclimation and/or passive heating interventions in people with neurodegenerative diseases will be addressed.

## Neurodegenerative Diseases

### Epidemiology and Pathophysiology

Central in the pathophysiology of neurodegenerative diseases is the loss of protein homeostasis and the progressive loss of selective neurons. Protein homeostasis involves a complex system of protein synthesis, folding, disaggregation, and degradation that ensures the correct function of the human body and particularly the central nervous system (Klaips et al., 2018). Loss of protein homeostasis, due to protein mis-folding and aggregation of damaged proteins, is a hallmark of neurodegenerative diseases such as Alzheimer’s and Parkinson’s diseases (Labbadia and Morimoto, 2015). Alzheimer’s and Parkinson’s are the two most common degenerative neurological conditions and are more prevalent with advancing age. Both of these neurodegenerative diseases are progressive with pathological features demonstrating topographic distribution. The progressive loss of selective neurons includes amyloidosis, tauopathies, alpha-synucleinopathies, and proteinopathies, all of which have their own characteristic histopathological imaging features, as well as clinical symptomology. The diseases are incurable and result in long-term cognitive, psychological, motor, and non-motor impairments that have a profound impact on functional mobility, psychological well-being, independent living, and quality of life.

#### Alzheimer’s Disease

Alzheimer’s disease is the most prevalent neurodegenerative disease and is the most common form of dementia (Thies and Bleiler, 2012), which affects 40–50 million people worldwide (Prince et al., 2013; Nichols et al., 2019). Early stages of Alzheimer’s presents with mild cognitive impairment involving memory loss and progresses with deficits in attention, language, and visuospatial abilities (Galton et al., 2000; Wattmo et al., 2016). Social withdrawal accompanies disease progression, as symptoms include a reduced capacity to perform activates of daily living, impaired executive function and judgment, along with disorientation (Wattmo et al., 2016). These outcomes have a significant impact on independence, quality of life, and years of life with a disability (Martyr et al., 2019). Furthermore, the economic cost of dementia is $968 billion globally (Xu et al., 2017). These costs are born by individuals and their caregivers, social health services, as well as public and private health care providers (Castro et al., 2010). Due to the aging population, the prevalence and impact of Alzheimer’s disease are anticipated to increase in the future (Prince et al., 2013; Nichols et al., 2019).

Neurodegenerative diseases such as Alzheimer’s are marked by a loss of cellular protein homeostasis (Ciechanover and Kwon, 2017; Klaips et al., 2018). The pathophysiology of Alzheimer’s is evidenced by intracellular and extracellular amyloid-β plaques as well as neurofibrillary tangles of hyperphosphorylated tau (Montine et al., 2012; Ciechanover and Kwon, 2017). Neurodegeneration occurs as a result of the accumulation of tau proteins and atrophy of cerebral cortices. Amyloid deposits occur in the neocortex and hippocampus (Phases 1 and 2), the striatum (Phase 3), the brainstem (Phase 4), and the cerebellum (Phase 5) (Montine et al., 2012). In concert with abnormal protein accumulation, the pathogenesis of Alzheimer’s disease may also involve vascular impairments leading to chronic cerebral hypoperfusion (de la Torre, 2004; Akinyemi et al., 2013; Sweeney et al., 2018). To combat these pathophysiological progressions, therapeutic interventions to improve protein quality control and regulation or improve vascular health and function have been recommended (Akinyemi et al., 2013; Ciechanover and Kwon, 2017).

#### Parkinson’s Disease

Parkinson’s disease is the second most common neurodegenerative disease, after Alzheimer’s disease. For Parkinson’s disease, the progressive degeneration of dopaminergic neurons in the substantia nigra pars compacta results in severe motor (e.g., tremor, rigidity, bradykinesia, postural instability) and non-motor symptoms (e.g., sleep disturbances, apathy, cognitive dysfunction, anxiety, depression) (Politis et al., 2010; Asahina et al., 2013). Both motor and non-motor impairments contribute to reduced physical activity and consequently decreased fitness in people with Parkinson’s disease (Speelman et al., 2011). This decreased fitness exacerbates both pre-existing and disease-specific conditions including cardiovascular disease, muscle weakness, postural instability, osteoporosis, sleep disruption, impaired cognitive function, depression and constipation (Speelman et al., 2011). Estimates suggest that Parkinson’s disease affects between 5 and 7 million people worldwide (Dorsey et al., 2018). The annual economic cost of Parkinson’s disease is estimated to be £3.3 billion in the United Kingdom (Findley, 2007), $23 billion in the United States (Findley, 2007), $6.3 billion in Australia (Access Economics (Firm) and Parkinson’s Australia, 2007).

The degradation in neural function in Parkinson’s disease is evidenced by degeneration of substantia nigral neurons, accumulation of alpha-synuclein and Lewy bodies, cortical atrophy and alteration in neural oscillatory activity between basal ganglia, thalamus, cortex, and brainstem areas. It has been proposed by Braak et al. (2003) that in Parkinson’s disease, the topographic progress of neurodegeneration, follows patterns of alpha-synuclein aggregation expressed in Lewy neurites and Lewy bodies. This occurs first in the medulla and olfactory bulb, then progressively dorsally through the brainstem, mediobasal forebrain, limbic structures, higher-order sensory association and prefrontal areas, and finally to primary sensory and motor areas (Braak et al., 2003). A central tenet of this proposal is that the initial disease onset is a result of inflammatory processes in the enteric system that result in progression to the central nervous system via the vagal nerve (Breen et al., 2019). Additionally, deterioration in the vasculature of the brain resulting in abnormal cerebral perfusion patterns has been identified in people with Parkinson’s disease, suggesting a role of cerebral blood flow in the pathophysiology of the disease (Melzer et al., 2011; Syrimi et al., 2017; Sweeney et al., 2018).

### Heat Shock Proteins as a Therapeutic Target

Recent reviews have clearly identified the upregulation of HSPs as thermally activated therapeutic targets for the treatment of neurodegenerative diseases including Parkinson’s and Alzheimer’s (Carman et al., 2013; Kalmar et al., 2014; Schapira et al., 2014; Ciechanover and Kwon, 2017; Webster et al., 2017; Klaips et al., 2018). HSPs are a collective family of proteins, suffixed by their molecular weight (in kilodaltons; kDa), which are present in both constitutively expressed, and inducible isoforms across several intracellular tissue sites and in extracellular fluid following stress (Kampinga et al., 2009). Relative to increased intracellular HSP content (a necessary component for protective cellular adaptation), the presence of extracellular changes in HSP concentration reflects a less pertinent (in the context of adaptation) transient stress response which acts as an acute signaling response. The 70 kDa (HSPA) and 90 kDa (HSPC) family of HSPs, hereafter referred to as HSP70 and HSP90, are generally the most widely studied responders to thermal stressors and are likely of most relevance within the field of heat therapy and heat adaptation for neurodegenerative disease (Luo et al., 2010; Fontaine et al., 2016; Lackie et al., 2017). HSP70 and HSP90 function as chaperones to ensure appropriate cell function and have distinct roles in the unfolded protein response, e.g., recognizing misfolded or mis-localized proteins that may be subsequently degraded by the proteasome, and are a key component of chaperone-mediated autophagy (Adachi et al., 2009; Stetler et al., 2010; Leak, 2014; Kampinga and Bergink, 2016; Zarouchlioti et al., 2018). It is outside of the focus of this review to describe each of these roles, with the reader directed elsewhere to contextualize these actions (Adachi et al., 2009; Stetler et al., 2010; Leak, 2014; Kampinga and Bergink, 2016; Zarouchlioti et al., 2018).

As therapeutic targets, HSP70 and HSP90 may be considered to have a direct and indirect role in neurodegenerative diseases. Direct roles for HSPs on the nervous system arise from the aforementioned notion that aggregation of misfolded proteins is characteristic of neurodegenerative diseases, including Parkinson’s, Alzheimer’s, and Huntington’s (Stetler et al., 2010). In Parkinson’s, HSP70 is reported as being of decreased gene expression (Mandel et al., 2005), while during proteomic profiling, reduced phosphorylation of HSP90 is also reported (Kulathingal et al., 2009). Pharmacological and animal models utilizing HSP expression (elevated HSP70 and reduced HSP90) have reduced the aggregation and toxicity of alpha-synuclein in Parkinson’s disease (Auluck et al., 2002; Klucken et al., 2004; Danzer et al., 2011; Gao et al., 2015). In Alzheimer’s, HSP70 may suppress the proteolysis of amyloid precursor protein (Hoshino et al., 2007) and in addition to HSP70, HSP90, and small HSPs reduce the formation of A-beta fibrils (Kudva et al., 1997; Lee et al., 2005; Wilhelmus et al., 2006a) and A-beta toxicity (Wilhelmus et al., 2006b) which subsequently form amyloid plaques. Tauopathy occurrence in Alzheimer’s may also be positively impacted by HSP changes with HSP70 (Jinwal et al., 2009) and HSP90 (Evans et al., 2006; Luo et al., 2007). Further, HSPs have been found to regulate huntingtin via reduced cell aggregation in Huntington’s disease (Muchowski et al., 2000; Sittler et al., 2001), and slows the muscle denervation of amyotrophic lateral sclerosis (Motor Neuron Disease) (Kieran et al., 2004; Kalmar et al., 2008, 2012).

Much of the literature describing these responses involve complex and isolated tissue/cell models to understand how HSP manipulation impacts upon neurodegenerative disease factors, thus direct application for humans remains unknown. However, with mechanistic support for the role of HSP augmentation to improve disease states, the application of heat therapy and/or heat adaptation in this context warrants further investigation.

## Responses to Active (Exercise Heat Acclimation) and Passive Heat Therapy in Healthy Adults

Physical activity and exercise have long been identified as mechanisms of inducing physiological stressors and subsequent positive adaptations in healthy (Tyler et al., 2016) and chronic disease (Hoffmann et al., 2016) populations. Unfortunately, those with increasing disease severity or diseases that challenge their motor control capabilities may be physically incapable of performing such beneficial exercise. Heat therapy has recently been targeted as a potential vehicle to evoke these positive thermal-induced adaptations in those precluded from undertaking exercise. Experimental investigations, large cohort surveys and reviews have expressed the potential for passive heating to improve physical and mental health in patients with cardiovascular disease (Brunt et al., 2016a, b; Maeda et al., 2018), diabetes (Kimball et al., 2018; Maley et al., 2019), peripheral arterial disease (Akerman et al., 2019), and depression (Janssen et al., 2016).

While there is a myriad of beneficial physiological and molecular effects of active and passive heating, this review will primarily focus specifically on the outcome of HSP expression, for its potential to influence proteostasis in neurodegenerative disease. For active and passive heating to be effective in increasing HSP expression, the minimum exposure requirements to elicit a desirable response in HSPs, from both acute and chronic (repeated) exposure, needs to be identified.

### Acute Effects of Body Warming on Heat Shock Proteins

Transcription of HSP mRNA, an essential step before protein translation, is primarily regulated by Heat shock factor protein 1 (HSF-1) as part of the Heat Shock Response (Kregel, 2002). HSF-1 activation involves a complex series of regulatory events, including nuclear localization, oligomerization and acquisition of HSE–DNA binding, ultimately resulting in the transcription of HSP mRNA (Sarge et al., 1993) in response to the thermal and physiological challenge (McClung et al., 2008; Maloyan et al., 2011). Sufficient mRNA transcription then leads to increased protein within the stressed cell.

Precise parameters for intracellular increases, and thus cellular adaptation, have been less clearly defined. For example, mean core body temperature may not be the sole marker of an increase, rather the rate of change in core body temperature may be of greater importance to signal HSF-1 to HSP70 pathways. In the more common exercise-heat stress model, a recent analysis concluded that when transcription of the related HSP70 and HSP90α mRNA is important, protocols should rapidly induce large, prolonged changes in core body temperature (Gibson et al., 2016). This notion was supported by evidence that, when analyzed collectively, significant predictors of the post-exercise change in HSP70 and HSP90α mRNA were the change in mean and peak core body temperature, and the duration core body temperature was ≥38.5°C (Gibson et al., 2016). It should be acknowledged that these data describe responses to exercise-heat stress, an intervention that is likely to be challenging to implement in clinical populations. Accordingly, passive heating via body warming may prove to be a more efficacious intervention.

The HSP response to localized or whole body warming has also been investigated. In passive heating models, increases in HSP70 and HSP90 mRNA have been evidenced as peaking 30 min following 90 min of local heating to either the thigh or the whole leg of healthy human volunteers (Kuhlenhoelter et al., 2016). Regrettably, no intramuscular temperature data is available from this experiment to assist with identifying minimum exposure requirement. This increase following resting, local heat stress which does not alter core body temperature offers mechanistic insight as elevations in blood flow and shear stress provide a non-core body temperature dependent HSP response that parallels transcription of angiogenic markers (Kuhlenhoelter et al., 2016). Not all experimental work has observed changes in HSP following passive heating. Leg immersion in hot water at 45°C for 60 min, eliciting an increased intramuscular temperature of >39°C, did not affect muscle HSPs (HSP70, HSC70, HSP60, HSP27, alphaB-crystallin) in healthy young humans (Morton et al., 2007). It should be noted that this null-observation came 48 h following, rather than immediately after heating, which may provide a rationale for the response. These data share commonality with responses observed elsewhere in relation to HSP70 and HSP27 stasis 24 h following ∼80 min of heating at ∼49°C (Vardiman et al., 2013) to increase intramuscular temperature also to >39°C, suggesting that the dose of heat therapy may be an important driver of HSP response or that the inconsistent timing of differential tissue sampling are experimental artifacts impacting current understanding.

Examination of extracellular changes in HSP70 during acute exercise-heat stress in humans has identified that the endogenous requirement for extracellular HSP70 release (at the cessation of exercise) may be a core body temperature mean of >38.5°C (peak of 39.2°C) for 56 min, alongside moderate exercising intensities (Gibson et al., 2014). Although changes may occur more rapidly (within 27 min) if exercise intensities are higher (Périard et al., 2012). Both the change in and final core body temperature attained are relevant to extracellular HSP70 release (Périard et al., 2012) and indicate achieving substantial elevations in thermal parameters is important when administering exercise-heat exposures to increase thermotolerance in whole-body models.

### Chronic Effects of Body Warming on Heat Shock Proteins

The HSP responses to exercise-heat acclimation have been reviewed previously, with an acknowledgment that the intervention is an effective means to augment cellular thermotolerance, which may subsequently protect vital organs from deleterious effects of heat stress in humans (Amorim et al., 2015). An internal temperature threshold for intracellular HSP70 induction may exist, though it is also possible that this response occurs once a certain variation of internal, whole-body temperature is reached alongside additional stressors (Magalhães et al., 2010). For example, during a 10-day heat acclimation period, Magalhães et al. (2010) demonstrated the largest changes in post-exercise intracellular HSP70 when a core body temperature >39.0°C was achieved. In contrast, Yamada et al. (2007) and Hom et al. (2012) reported no change in HSP70 in response to a lower core body temperature (mean maximum of ∼38.5°C) following 10 days of heat acclimation. HSP70 and HSP90 mRNA transcription occur at a series of core body temperature thresholds during 90 min of exercise-heat stress (mean 37.6–38.2°C; peak 38.1–39.1°C) (Gibson et al., 2015a, b) therefore the dose of heat stress to elicit translation may be greater than that required to elicit transcription.

In human whole-body passive heating models, such as hot water immersion of >60 min whereby core body temperature is increased, higher extracellular HSP70 concentration (Faulkner et al., 2017), and intracellular HSP70 changes (Oehler et al., 2001), have been reported. The beneficial response once again is not unanimous, with others reporting chronic change (following 2 weeks of repeated therapy) in intracellular HSP70 after 45–60 min of passive heat therapy (Hoekstra et al., 2018). In a similar manner to that described for local heating, the dose of stress may be important given the lack of change in studies using 60 min heating (Hoekstra et al., 2018), in comparison to those who do report an acute increase in intracellular HSP70 following 120 min heating in 39°C water (Oehler et al., 2001).

Intracellular HSP70 and HSP90 levels in peripheral blood mononuclear cells (PBMC) are increased after 6–10 days of long term exercise-heat acclimation (Yamada et al., 2007; McClung et al., 2008). The two larger HSPs appear to be related with regards to exercise-heat stress changes, as an HSP70 increase of ∼21% was correlated with HSP90 increases of ∼18% (McClung et al., 2008). *In vitro* analysis of PBMC obtained from 10-day exercise-heat acclimated individuals exhibit greater blunting of the HSP response to heat shock of 43°C for 60 min (compared to unacclimated). This blunted pre-post response is indicative of increased thermotolerance and/or cellular protection from stress, likely due to increased basal intracellular HSP content and appears directly related to the degree of physiological heat acclimation (lower core temperature), thus the adapted individual/cell experiences lesser relative stress at the same absolute temperature (McClung et al., 2008).

The notion of individual differences in the responses to local heat therapy has been highlighted in a study observing that 24 h following 40 min of heat treatment (diathermy followed by heat packs), female subjects significantly increased HSP70 (+58%) and phosphorylation of HSP27 (+100%) content compared to the untreated leg (Touchberry et al., 2007). In comparison, male subjects had non-significant increases in HSP70 (+35%) and HSP27 phosphorylation (+32%) within skeletal muscle (Touchberry et al., 2007). These sex-specific responses are intriguing and warrant future investigation given no differences in Hsp70 mRNA have been reported during isothermic heat acclimation (Mee et al., 2016) and were not reported in the mixed-sex cohort undertaking passive heating described above (Kuhlenhoelter et al., 2016).

### Central and Peripheral Effects of Body Warming

The administration of heat therapy and heat acclimation may provide additional benefits in the context of neurodegenerative diseases in relation to skeletal muscle function, cerebral blood flow, and metabolic health. Adverse reductions in strength and lean body mass are symptoms of neurodegenerative diseases, including Alzheimer’s disease (Burns et al., 2010; Buchman and Bennett, 2011), Parkinson’s disease (Berardelli et al., 2001; Petroni et al., 2003; Cano-de-la-Cuerda et al., 2010) and Amyotrophic Lateral Sclerosis (Gubbay et al., 1985; Munsat et al., 1988; Kiernan et al., 2011). While various mechanisms are at play, muscle atrophy and decreased strength likely owe to symptom-influenced reductions in physical activity, along with central and peripheral nervous system changes that limit muscle activation (Hass et al., 2007). Furthermore, reduced cerebral blood flow and poor metabolic health profiles may also be related to disease progression (de la Torre, 2004; Akinyemi et al., 2013; Bharadwaj et al., 2017; Sweeney et al., 2018). As there is a potential benefit to be gained, the below reviews the current developing understanding of the acute and chronic effects of elevated temperature on skeletal muscle function, cerebral blood flow, and markers of metabolic health.

#### Skeletal Muscle Function

Increases in skeletal muscle temperature have long been accepted to improve acute muscle force, power and contractility (Bergh and Ekblom, 1979; Davies and Young, 1983; Bennett, 1984). Contrastingly, the inverse relationship between high core body temperature and muscle torque, muscle recruitment patterns, and voluntary activation is also documented (Morrison et al., 2004; Todd et al., 2005; Thomas et al., 2006). Importantly, however, these outcomes are most often viewed with a short-term lens, with limited understanding of the effects of time or repeated heat exposures on skeletal muscle in humans (Brazaitis and Skurvydas, 2010; Goto et al., 2011; Racinais et al., 2017).

Passive heating has been reported in experimental designs examining effects on exercise-induced muscle damage (Nosaka et al., 2007; Touchberry et al., 2012), recovery from muscle injury (Kojima et al., 2007; Oishi et al., 2009; Takeuchi et al., 2014) and immobilization (Selsby and Dodd, 2005; Senf et al., 2008; Dodd et al., 2009), and muscle hypertrophy (Uehara et al., 2004; Ohno et al., 2010) in animal models. The rationale for passive heat application relates to the altered cascade of inflammation and HSP expression that interact with mitochondrial biogenesis and muscle growth (Yoshihara et al., 2013; McGorm et al., 2018). Increases in wet muscle mass and protein content in rat soleus muscle have been described 7 days after a 60 min exposure to a 41–42°C heat chamber (Uehara et al., 2004; Ohno et al., 2010). Further, Kodesh and Horowitz (2010) observed higher muscle mass/body weight ratios in rats following 30 days of acclimation to 34°C environmental heat compared to a 24°C control. Similarly, in healthy men, Goto et al. (2011) saw an increased cross-sectional area of fibers in the vastus lateralis (8.3%) using a steam-generating sheet applied to the quadriceps muscle for 8 h⋅day^–1^ and 4 days⋅week^–1^ across a 10-week intervention. Collectively, it might be concluded that passive heating could support cell proliferation and facilitate muscle hypertrophy (Naito et al., 2000; Goto et al., 2004; Uehara et al., 2004). Such outcomes would be particularly beneficial to those experiencing neurodegenerative disease, particularly as passive heating appears also to attenuate human skeletal muscle atrophy (Hafen et al., 2019).

Most pertinent from a translational perspective, improved strength has been demonstrated to couple the increased skeletal muscle growth after passive heating (Goto et al., 2011; Racinais et al., 2017). Higher isometric knee extensor torque (5.8%) was achieved after 10 weeks of heat stress, which the authors explained as potentially relating to the increase of myonuclear number (Goto et al., 2011). Goto et al. (2011) also found a 4% increase in knee extensor strength in the non-heated leg. While the contralateral effects of unilateral resistance training are acknowledged (Lee and Carroll, 2007; Frazer et al., 2018), this phenomenon may imply that there are central nervous system effects of chronic passive heating. A potential role of circulating factors has also been proposed (Hendy and Lamon, 2017). The site(s) of possible neural adaptation explaining cross-education remain unclear. However, it is conceivable that adaptations could occur at the spinal and/or cortical level considering the noted decline in descending motor drive during acute episodes of hyperthermia (Todd et al., 2005; Périard et al., 2012; Ross et al., 2012). It seems that higher body temperature acutely impairs somatosensory processing (Nakata et al., 2015), though how adaptation to passive heating might affect neural activity in healthy or diseased states in thermoneutral conditions remains to be elucidated. Regardless, adaptation to passive heat therapy is promising, particularly in a rehabilitation setting and for those with neurodegenerative diseases, as acute stress may increase motor cortex excitability and augment motor skill acquisition (Littmann and Shields, 2016).

#### Cerebral Blood Flow

Another avenue by which passive heating may have a therapeutic effect is through improved cerebral blood flow. Reduced cerebral blood flow and dysfunction in the blood-brain barrier have been identified in neurodegenerative diseases, including Alzheimer’s and Parkinson’s (de la Torre, 2004; Akinyemi et al., 2013; Sweeney et al., 2018). Both motor and cognitive impairments have been associated with poor perfusion in several brain regions in Parkinson’s disease (Melzer et al., 2011). Similarly, overall and regional cerebral blood flow reductions have been associated with cognitive decline in mild cognitive impairment and Alzheimer’s disease (Leeuwis et al., 2017). For these reasons, the vasculature supplying blood to and across the brain are also relevant targets to examine the beneficial adaptations of passive heat therapy for neurodegenerative diseases.

Several vascular adaptations attributable to heat therapy have been reported. These have included improved flow-mediated dilation (Brunt et al., 2016a), increased pulse wave velocity (an index of arterial stiffness) (Brunt et al., 2016a), reduced carotid intima thickness (Brunt et al., 2016a) improved capillarization (Hesketh et al., 2019) and subsequently enhanced systemic blood pressure profiles (Brunt et al., 2016a; Akerman et al., 2019). Mechanistically, HSP27 has been shown to reduce intimal hyperplasia (Connolly et al., 2003), with greater carotid intima thickness associated with reduced cerebral blood flow (Sojkova et al., 2010). Undesirable vascular hypertrophy can also be mitigated against via HSP70 associated inhibition of Angiotension II (Zheng et al., 2006), with HSP90 conferring a more general adaptation aligned to the stabilization of the vascular endothelial growth factor (VEGF) upstream target hypoxia-inducible factor-1 (HIF-1α) (Maloyan et al., 2005), and elevated endothelial nitric oxide production and improved the stabilization and bioavailability of endothelial nitric oxide synthase (Averna et al., 2008). Collectively these vascular adaptations observed in passive heating interventions have the potential to maintain cerebral blood flow and blood-brain barrier function, outcomes which may have beneficial effects for cognitive function in Alzheimer’s and Parkinson’s diseases.

#### Metabolic Health

Markers of metabolic health may also play a role in the pathogenesis of neurodegeneration in Alzheimer’s disease. The neurodegeneration seen in Alzheimer’s disease has been linked with impaired cerebral insulin signaling and glucose metabolism (Bharadwaj et al., 2017). The loss of protein homeostasis (Aβ accumulation and tau hyperphosphorylation), synaptic degeneration, and neural dysfunction have been associated with these impairments to normal metabolic processes (Bharadwaj et al., 2017). Recent research has therefore proposed therapeutic interventions to improve insulin signaling and reduce insulin resistance. Interestingly, heat therapy has been proposed for people with type II diabetes, and early studies have highlighted a reduction in fasting plasma glucose following repeated hot water bath immersions over 3 weeks (Hooper, 1999). Furthermore, chronic heat therapy interventions have improved glucose tolerance and insulin sensitivity in women with polycystic ovary syndrome who experience obesity and metabolic dysfunction (Ely et al., 2019). Therefore, the effects of heat therapy on metabolic health are another potential avenue of therapeutic benefit for people with neurodegenerative disease.

### Summary

**FIGURE 1:**
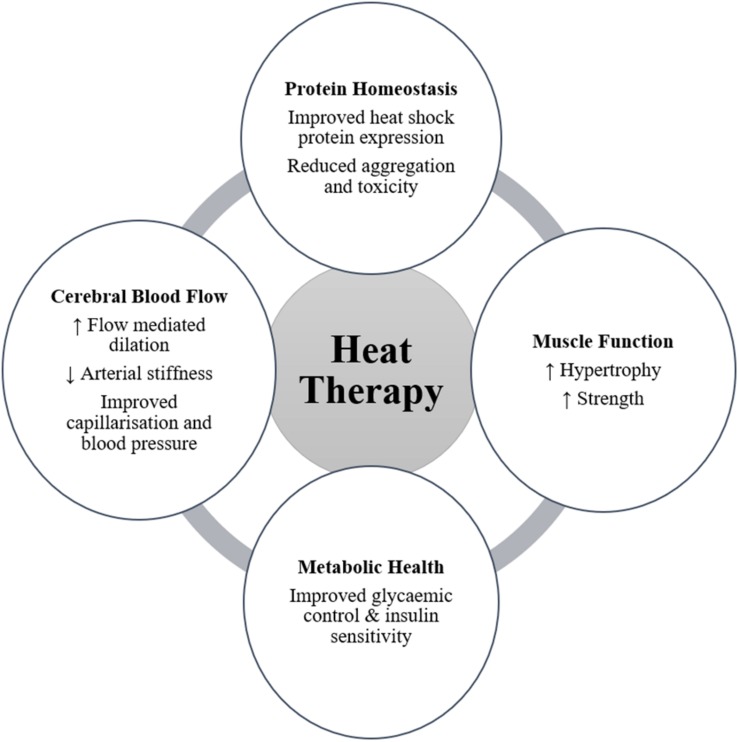
Potential benefits arising from heat therapy for people with neurodegenerative disease (↑ symbolizes an increasing effect; ↓ symbolizes a decreasing effect).

For individuals who do not experience these important physiological stressors through habitual activity and exercise, heat therapy may provide a vehicle to achieve a range of health and physiological benefits (Figure 1). Much remains to be understood with regards to the mechanisms and stimuli required to elicit the desired increases in relevant HSPs following heat therapy, in addition to further quantifying the magnitude of importance of these responses. Additionally, the potential to improve muscular function, cerebral blood flow, and markers of metabolic health offer significant benefit for people with a neurodegenerative disease by improving their quality of life and reducing disease severity. Part of the present ambiguity results from the utilization of different methods, e.g., heating technique, heating duration and magnitude, tissue sample site and time points, across experimental studies. Despite the need for further clarity regarding the mechanistic underpinnings and best practice implementation, the opportunities to investigate a tolerable heat therapy model in a relevant target population exists and should be encouraged.

## Body Warming in People With Neurodegenerative Diseases

### Evidence for a Potential Benefit

At present, the authors are not aware of any studies that have directly assessed the effects of active or passive body warming on HSP release, and disease severity or progression, in people with neurodegenerative diseases. Therefore, this review will draw on findings from epidemiological studies, and indirect studies of other interventions such as exercise, that have shown benefits among people with neurodegenerative diseases.

Habitual body warming, through sauna bathing or exercise, has been shown to reduce the risk of developing neurodegenerative diseases. Regular passive heating has been associated with a reduced risk of developing neurodegenerative diseases, including Alzheimer’s (Heinonen and Laukkanen, 2018). Men participating in sauna bathing 2–3 or 4–7 times per week had a 0.80 and 0.35 hazard ratio for developing Alzheimer’s disease compared to men who sauna once per week or less (Laukkanen et al., 2017). Regular exercise also has a protective effect on the risk of developing Alzheimer’s and Parkinson’s diseases (Paillard et al., 2015). Consistent and frequent participation in moderate to vigorous physical activity was found to reduce the risk of Parkinson’s disease by up to 40% (Xu et al., 2010). Similarly, exercising three or more times per week is associated with a lower incidence rate of dementia and Alzheimer’s (Larson et al., 2006). While these studies do not elucidate the potential mechanisms by which a protective effect is elicited, it does lend anecdotal evidence toward the expression of HSP through regular body warming as a distinct possibility.

Further to a preventative effect, exercise has been described as having a restorative effect on the neurodegeneration observed in Alzheimer’s and Parkinson’s diseases (Loprinzi et al., 2013; Paillard et al., 2015). Clinical and epidemiological studies have provided evidence supporting the conclusion that exercise has therapeutic value by reducing the symptoms and slowing disease progression (Ransmayr, 2011; Hou et al., 2017; Liu et al., 2019). Moderate to high-intensity aerobic exercises, such as treadmill walking or assisted cycling, are recommended for improved motor and cognitive function in Parkinson’s disease (Ahlskog, 2011; Salgado et al., 2013; Evens and Clark, 2017). High-intensity treadmill exercise of 30 min at a target heart rate prevented Parkinson’s disease progression, compared to moderate intensity and control (Schenkman et al., 2018). Furthermore, interval exercises (alternating periods of low and high intensity assisted cycling) have shown positive improvements in functional ability in Parkinson’s patients (Uygur et al., 2015, 2017). Immersion in warm water (33°C) for 50 min while performing dual-task exercises (combining physical movements with cognitive tasks) was found to improve functional mobility (timed up and go, and five-time sit-to-stand) among people with Parkinson’s disease following 3 months of twice-weekly exposures (Silva and Israel, 2019).

Exercise interventions have also had significant effects on slowing the progression of Alzheimer’s disease. Over 1 year, people with Alzheimer’s disease who participated in twice-weekly 1-h exercise sessions showed reduced rates of decline in measures of functional independence and physical performance, compared to control participants (Pitkala et al., 2013). In a similar intervention, the ability to perform activities of daily living declined significantly slower in people with Alzheimer’s disease performing twice-weekly exercise programs (Rolland et al., 2007). A 6-month walking program has also shown people with Alzheimer’s disease to be able to maintain cognitive function on the Mini Mental State Exam (MMSE), compared to significant declines in those not exercising (Venturelli et al., 2011). Overall, there is compelling evidence that exercise improves motor and cognitive function in neurodegenerative disease and it is therefore recommended by clinicians (Ransmayr, 2011; Hou et al., 2017; Liu et al., 2019). However, exercise and body warming interventions should consider the difficulties in performing such activities for these populations (section “Considerations for Heat Therapy for People With Neurodegenerative Disease”).

The studies of moderate to high-intensity exercise among neurodegenerative disease populations provide circumstantial evidence that the assumed body warming experienced may be contributing to the beneficial effects observed. While there may be many avenues by which exercise and body warming promote improved health and function, the role of body temperature elevation, thermoregulatory responses, and HSP expression have been overlooked in these experiments. Given the growing body of evidence that supports the expression of HSPs as therapeutic targets for Alzheimer’s and Parkinson’s diseases (section “Heat Shock Proteins As a Therapeutic Target”), there is a clear need for future investigations of passive heating to monitor thermoregulation and HSP responses in people with these neurodegenerative diseases.

### Considerations for Heat Therapy for People With Neurodegenerative Disease

An important consideration in conducting heat acclimation and heat therapy for older adults and clinical populations will be how their impairments or any co-morbidities may affect their ability to perform and tolerate these interventions. Firstly, their disease severity may impair their physical ability to perform movements effectively and safely (Kerr et al., 2010; Buchman and Bennett, 2011). Secondly, neurodegeneration may cause deficits in thermoregulatory processes. In Parkinson’s disease, neural degeneration in higher-order brain centers including the hypothalamus is associated with impaired sudomotor function which in turn may influence their tolerance to body warming (LeDoux, 2013).

Neurodegenerative diseases such as Parkinson’s and Motor Neuron Disease primarily affect motor control. As the diseases progress, motor function deteriorates resulting in impaired gait and balance and an increased risk of falls (Pickering et al., 2007). While Alzheimer’s is usually associated with cognitive impairments, significant motor impairments are also associated with this disease (Buchman and Bennett, 2011). Therefore, exercise and heat acclimation interventions should consider the level of impairment of their target population and how the risk of falls and injury may be managed. As such, in these populations where movement is limited, passive heat therapy may be an achievable alternative to exercise interventions.

The autonomic nervous system, responsible for thermoregulation, can exhibit deficits in neurodegenerative diseases, specifically in the thermoeffector responses of sweating and skin blood flow. Abnormalities in the sweating response, hyperhidrosis and/or hypohidrosis, are commonly reported in Parkinson’s disease (De Marinis et al., 1991; Swinn et al., 2003; Schestatsky et al., 2006) and may be more prominent with increasing age and disease severity of patients (Saito and Kogure, 1989). Hypohidrosis, an absence of the sweating or reduced sweat output, may reduce effective body cooling during exercise and body warming. However, the sweating response is highly individualized, and compromised sweating in one body region may be compensated by increased sweating in other body regions (Schestatsky et al., 2006). Careful attention should, therefore, be given to the rate of body warming people with Parkinson’s disease may experience, and ensuring appropriate cooling strategies are available.

Cardiovascular regulation of blood pressure is also influenced by neurodegenerative disease. The expected elevation in heart rate and blood pressure may be blunted in people with Parkinson’s disease, and they may also experience post-exercise hypotension (Asahina et al., 2013). Orthostatic intolerance is also reported in 10–60% of people with Parkinson’s disease (Senard et al., 1997; Wüllner et al., 2007). These cardiovascular impairments may influence their ability to tolerate exercise and body warming and should be considered in determining individual suitability for heat therapy and heat acclimation. The modality of passive heating may therefore also be an important factor in determining appropriate therapeutic techniques. Infrared sauna bathing has been reported to promote lower cardiovascular strain than traditional sauna techniques (Mero et al., 2015) and may be one avenue that could be investigated for suitability in these at-risk populations.

## Future Directions

Overall, there are three key points from the scientific literature that support a proposal for a therapeutic effect of heat therapy or heat acclimation to promote HSP expression in people with neurodegenerative disease. These include, (1) Exercise, sufficient to raise core body temperature, is currently recommended for people with neurodegenerative disease (e.g., Parkinson’s and Alzheimer’s diseases) as it has been shown to improve their symptoms; (2) Elevated HSP levels have been identified as a therapeutic target to reduce protein aggregation and toxicity; and (3) Exercise and body warming have been shown to elevate HSP expression in healthy adults. Furthermore, heat therapy may have additional benefits for muscle function, vasculature health and cerebral blood flow, and indicators of metabolic health, which have also been implicated in the pathophysiological presentation of neurodegenerative diseases. These findings from the current scientific literature support the proposal for further investigation into the potentially beneficial adaptations for people with neurodegenerative diseases to heat therapy and heat acclimation.

Initial research is required to establish the acute effects of heat therapy and/or heat acclimation in people with neurodegenerative diseases. As these diseases may involve impairment of autonomic pathways involved in thermoregulation, research is required to elucidate the thermoeffector responses, sweating and skin blood flow, to acute heat stress and how these may affect tolerance to body warming. Alongside these outcomes HSP expression, muscular adaptations, and vasculature function responses to body warming should be measured in neurodegenerative disease populations, to determine the presence of a similar response to body warming as seen in healthy adults, and the magnitude of the response in relation to the tolerable limits of body warming. Consequent to acute observations that tolerable heat exposures promote the desirable HSP response and vascular adaptations, further investigation should then pursue the effects of chronic or repeated heat therapy and/or heat acclimation on these responses and indicators of disease severity and progression. Finally, an understanding of the dose-response relationship between frequency and intensity of body warming and improved symptomology should be determined. In concert with these research efforts, investigations of the underlying mechanisms by which HSP expression and body warming may improve neuromuscular function are warranted.

## Conclusion

While the current evidence for heat therapy in neurodegenerative disease is associative and the mechanisms of improved health outcomes have yet to be elucidated, the potential of passive heating in this population remains an alluring therapeutic option. Heat acclimation and heat therapy have been shown to improve HSP responses in healthy adults, as well as induce additional benefits for skeletal muscle function and cerebral blood flow. Furthermore, HSPs have been identified as therapeutic targets to restore protein homeostasis and reduce protein toxicity in several neurodegenerative diseases. While circumstantial evidence exists that body warming may promote improved health and function in neurodegenerative disease, through exercise or sauna bathing, there is yet to be any direct studies of body warming, thermal tolerance, and HSP responses in these populations. For individuals who do not experience these important physiological stressors through habitual activity and exercise, heat therapy may provide a vehicle to achieve improved health and slowed disease progression in people with neurodegenerative disease, and, therefore, warrants further investigation.

## Author Contributions

All authors contributed to the planning and writing of the manuscript. Specifically, AH contributed to the design, writing, and review of the manuscript and was the primary contributor to section “Body Warming in People With Neurodegenerative Diseases.” GM contributed to the design, writing, and review of the manuscript and was the primary contributor to section “Central and Peripheral Effects of Body Warming.” OG contributed to the writing and review of the manuscript and primarily contributed to sections “Heat Shock Proteins As a Therapeutic Target,” “Acute Effects of Body Warming on Heat Shock Proteins,” and “Chronic Effects of Body Warming on Heat Shock Proteins.” GK contributed to writing section “Epidemiology and Pathophysiology.” IS contributed to the overall plan and design of the manuscript and writing sections “Introduction” and “Future Directions.”

## Conflict of Interest

The authors declare that the research was conducted in the absence of any commercial or financial relationships that could be construed as a potential conflict of interest.
